# Individual differences in emotion regulation and face recognition

**DOI:** 10.1371/journal.pone.0243209

**Published:** 2020-12-10

**Authors:** Ahmed M. Megreya, Robert D. Latzman

**Affiliations:** 1 Department of Psychological Sciences, College of Education, Qatar University, Doha, Qatar; 2 Department of Psychology, Georgia State University, Atlanta, GA, United States of America; Bournemouth University, UNITED KINGDOM

## Abstract

Face recognition ability is highly variable among neurologically intact populations. Across three experiments, this study examined for the first time associations between individual differences in a range of adaptive versus maladaptive emotion regulation strategies and face recognition. Using an immediate face-memory paradigm, in which observers had to identify a self-paced learned unfamiliar face from a 10-face target-present/ target-absent line-up, Experiment 1 (*N* = 42) found high levels of expressive suppression (the ongoing efforts to inhibit emotion-expressive behaviors), but not cognitive reappraisal (the cognitive re-evaluation of emotional events to change their emotional consequences), were associated with a lower level of overall face-memory accuracy and higher rates of misidentifications and false positives. Experiment 2 (*N* = 53) replicated these finding using a range of face-matching tasks, where observers were asked to match pairs of same-race or different-race face images taken on the same day or during different times. Once again, high levels of expressive suppression were associated with a lower level of overall face-matching performance and higher rates of false positives, but cognitive reappraisal did not correlate with any face-matching measure. Finally, Experiment 3 (*N* = 52) revealed that the higher use of maladaptive cognitive emotion regulation strategies, especially catastrophizing, was associated with lower levels of overall face-matching performances and higher rates of false positives. All told, the current research provides new evidence concerning the important associations between emotion and cognition.

## Introduction

Face recognition ability is highly variable among neurologically intact populations [e.g., for reviews see 1–3]. On the one hand, individuals with developmental prosopagnosia have severe face recognition deficits in the absence of any brain damages [for reviews, see 4, 5]. On the other hand, individuals with extra-ordinary face recognition ability, oftentimes called super-recognizers, are able to perform rather challenging face recognition tasks with extremely high levels of accuracy [e.g., for reviews see 6, 7]. Between these two extremes, face recognition ability of the vast majority of neurologically intact individuals is distributed along this spectrum.

Across a set of studies involving a total of 400 participants, Woodhead and Baddeley (1981) noticed that *d*’, the sensitivity index in the signal detection theory, ranges from 0.5 to 6.8 using a facial recognition memory task (making old/new decisions for a set of previously studied faces or distractors) [8]. These wide individual differences were also noticed using face perception tasks, which do not rely on memory. For example, face-matching performance has generally been found to range between 50% to 96% accuracy [9] using an array task (matching a target unfamiliar face to a 10-face target-present/ target-absent lineup [10]. Further, using the Glasgow Face Matching Test (GFMT), a test that asks participants to match the identity of pairs of unfamiliar faces, individual performance ranges along a broad continuum from just above chance to perfect [11]. Importantly, not only are these individual differences reliably found across studies and tasks, they appear to be stable [12], unrelated to general intelligence [13], and highly heritable [14]. Therefore, understanding the processes associated with the individual differences in face recognition is a topic of interest [e.g., for reviews see 1–3].

### Demographic-related differences in face recognition

The vast majority of studies that have considered contributors to variation in facial recognition ability have largely focused on demographic characteristics of participants and face stimuli including race, age, and gender. For example, an own-race advantage has been well-documented such that individuals recognize faces belonging to their own-race more accurately than those belonging to other races [15–18]. In addition, developmental studies have reported that face recognition improves with development before deteriorating with old age [19, 20]. Furthermore, gender differences have been observed in face recognition, with an own-gender bias reliably found, especially for females [21–24]. Importantly, however, despite these demographic-related differences, face recognition ability still remarkably varies widely, even within demographically-homogeneous groups of individuals (i.e., within individuals belonging to the same race, age, and gender).

### Individual differences among face recognition tasks

Even within demographically-homogeneous groups, a great deal of variation in face recognition abilities exists. With this in mind, a general face recognition factor (termed *f)* has been proposed in order to explain this variation. For example, Verhallen et al. (2017) suggested that individuals who are good in a task measuring specific aspects of face perception are also good in other tasks measuring different aspects of face memory [25]. Specifically, Verhallen et al. (2017) reported positive inter-correlations among three standardized tests measuring different aspects of face recognition [25]. These included the GFMT, the Cambridge Face Memory Test (CFMT), a measure of face memory that requires participants to recognize sets of previously learned faces through three-alternative forced choice tasks, and the Mooney Face Test, a measure of face closure (a perceptual tendency to view incomplete objects as complete) that requires observers to identify the gender of faces using high-contrast images consisting of exclusively dark or light regions [25].

Similar findings have also been reported by McCaffery, Robertson, Young, and Burton (2018) who found positive inter-correlations among the GFMT, the CFMT, and the Before They Were Famous task (BTWF), a measure of familiar face recognition that requires observers to recognize a set of celebrities using photos taken before they became famous; when they were children or adolescents [26]. Robertson, Black, Chamberlain, Megreya and Davis (2020) also found positive and relatively strong inter-correlations between matching and memory tasks, which included same-race and other-race faces [27]. Stacchi, Huguenin-Elie, Caldara, and Ramon (2020) reported similarly strong positive correlations among a variety of face recognition tasks [28].

Further, matching upright unfamiliar faces has been found to positively correlate with recognition memory [9], immediate memory (identifying a learned face through a subsequent target-present/ target-absent 10 face line-up) [29], eye-witness identification (identifying a culprit eye-witnessed during a staged crime through a target-present/ target-absent line-up) [30], and matching inverted familiar and unfamiliar faces [9]. Further underscoring the replicability, across assessment modalities, of positive associations among distinct face-related tasks, performance on the CFMT positively has been found to correlate with the fast periodic visual stimulation (FPVS) paradigm, an objective EEG index of individual face discrimination in the right occipitotemporal cortex [31].

### Individual differences in face recognition and visual processing

In addition to face-related abilities more specifically, individual differences in face recognition also appear to co-vary with variation in visual processing abilities more generally. For example, almost forty-years-ago, Woodhead and Baddeley (1981) found that people who were good at recognizing faces were also good at recognizing other non-face visual objects [8]. More recently, Megreya and Burton (2006) found that performance on the 1-in-10 face matching task positively correlated with visual short term memory, perceptual speed (Finding A’s and Identical Picture Tests), and Matching Familiar Figure Test, a measure of object matching that requires participants to match a target line drawing of common objects to a line-up of six minor variants [9]. Similarly, performance on the GFMT positively correlated with the Matching Familiar Figure Test [11, 26] and with the Navon local processing task, a measure of parts perception that require observers to identify the identity of the small parts of the compound letters [26] as has performance on CFMT and the Cambridge Car Memory Test have also been found to be positively correlated with each other [32]. Performance on recognition memory for faces and visual processing speed in infants [33] and with visual perceptual speed in 11 year-old children [34] have similarly been found to positively associate with each other.

Although studies have consistently found face recognition to be affected by several visual processing skills, some studies have reported some distinctions between these two domains. For example, McCaffery et al. (2018) found that performances on the GFMT, CFMT and BTWF did not correlate with performances on a range of visual perception tasks including position discrimination (identifying which squire includes a more precisely central dot), position of gap (identifying whether the gaps in two circles are in the same or different positions), letter detection (detecting the letter “f” from a passage), and Navon global processing (identifying the identity of the large compound letters) [26]. Consistently, Wilhelm et al (2010) found that found that individual differences in three main aspects of face recognition (face perception, face memory, and speed of face cognition) could not be reduced to individual differences in immediate and delayed memory, general cognitive ability, mental speed, and object recognition [13]. Therefore, although face recognition is correlated with some visual processing tasks [9, 32], a consistent empirical literature provides support for the face-specific domain theory [35] suggesting that faces are processed through cognitive and neurological mechanisms that are not involved in object recognition [13].

### Individual differences in face recognition and personality and emotions

Intriguingly, although findings have been mixed, individual differences in face recognition seem to also relate to other “non-visual” processes including personality and emotions. For example, some studies have found that observers who exhibit high levels of extraversion and emotional stability are more accurate at face recognition than individuals with lower levels of these traits [36, 37]. Cheung, Rutherford, Mayes, and McPartland (2010) further found that those with higher in extraversion exhibited a discrepant N170 neurophysiological event-related potential amplitude, a face-specific brain electrophysiology component, associated with face inversion that was more prominent in the right hemisphere [38]. However, whereas neuroticism (i.e., low emotional stability) appears to be associated with a reliable negative impact on face identification [39], results from studies on extraversion and related traits (e.g., shyness [reverse-keyed] have been more mixed [40–43]. For example, Megreya and Bindemann (2013) examined the relationship between individual differences in performance in the 1-in-10 face matching task and a range of personality factors and found that correct face identifications related to low anxiety, low tension, and high emotional stability (i.e., low neuroticism) [44]. Lander and Poyarekar (2015) examined the relationship among upright/ inverted familiar face recognition (naming a set of British and American celebrities), the GFMT, and a brief measure personality revealed only one significant association: extraversion positively correlated with recognition of familiar faces when presented upright but not upside down; no other associations between face-recognition and personality emerged [45]. Further complicating the picture, McCaffery et al (2018) more recently found no relationship between the GFMT, CFMT and BTWF and personality [26].

One potential explanation for the mixed literature concerning associations between individual differences traits and face recognition may be variation in not only traits (i.e., general tendencies to experience various emotions) but also the way in which individuals regulate those emotions. Indeed, emotions generally, and emotion regulation more specifically, may play some roles on individual differences in face recognition. In support of this possibility, an early study reported that participants in a moderate arousal condition had higher face recognition ability than those in the high arousal condition [46]. More recently, Hills et al (2019) examined how being observed affects face recognition and found that being observed during learning, but not during the test phase, impaired recognition accuracy using an old/new recognition memory and eyewitness identification paradigms [47]. Although being observed was associated with increased physiological arousal as indexed by galvanic skin response and heart rate, these authors found that this heightened arousal did not explain the detriments in face recognition [47]. Nevertheless, face recognition has been found to negatively associate with generalized anxiety [44, 48–51]; but for an inconsistent finding see [52] as well as social anxiety more specifically [53]. In addition, observers scoring high on socio-emotional empathy also appear more accurate at face recognition than people who display lower levels of empathy [54]. Furthermore, the Matching Familiar Figures Test, which was initially developed as a measure of impulsiveness in children, has been shown to predict accuracy in face matching tasks [9, 11].

To summarize, there are wide individual differences in face recognition [e.g., for reviews see 1–3] and these individual differences are stable across different face perception and recognition tasks [25–28]. There have been many factors identified in the literature that may help to explain this variation including: (i) demographic characteristics including race [15–18], age [19, 20], and gender [21, 24]; (ii) a range of visual cognitive abilities such as general memory [8, 13, 32], object perception [11, 26], and perceptual speed [9, 33, 34]; (iii) personality traits such as extraversion [36–38, 45], neuroticism/low emotional stability [36–39, 44], impulsiveness [9, 11, 26], and socio-emotional empathy [54]; and (iv) anxiety-related symptomatology including generalized anxiety [44, 48–51], social anxiety [53], physiological arousal [47].

### Emotions and emotion regulation

It is well-established that emotions can be substantially modified using a range of emotion regulation (ER) strategies [e.g., for an extensive review see 55] and some scholars have even argued that both emotions and ER might be one process [56, 57]. ER has been conceptualized as “the processes or strategies through which individuals can modulate or manage which emotions they have, when they have them, and how these emotions are experienced and expressed” [58]. The widely-used process model of ER [58, 59] includes two broader types of ER strategies: 1) antecedent-focused strategies, referring to things people do before emotional response tendencies have completely activated and 2) response-focused strategies, referring to things people do once emotions have already been generated. In addition to these two broad types of strategies, more specific strategies are thought to fall along a temporal sequencing of emotion regulation processes. In addition, Garnefski, Kraaij, and Spinhoven (2001) provide a second widely-used model of ER in which a distinction is made between cognitive (e.g., making plans) and behavioral (e.g., taking immediate action) ER strategies [60].

A meta-analysis found that anxiety, depression, eating, and substance-related disorders were negatively associated with two adaptive ER strategies (problem-solving and reappraisal) and positively associated with three less-adaptive ER strategies (rumination, avoidance, and suppression) [61]. In addition to associating with various psychopathological outcomes, ER strategies appear to also have cognitive correlates. For example, Richard and Gross (2000) found that suppression impaired memory, but reappraisal had no effect [62]. Consistently, subsequent studies reported associations between ER and working memory [63–65]. For example, Schmeichel et al (2008) found that the successful suppression of facial expressions of emotions negatively correlated with working memory capacity [65]. In addition, McRae et al (2012) found a positive correlation between individual differences in reappraisal ability and working memory capacity [64]. Therefore, some studies have found that training working memory could improve ER strategies [66–68]. Notably, significant interactions between face recognition and working memory have been consistently found in behavioral and neuro-physiological experiments [69–71]. For example, activity in fusiform face area was found to modulate as a function of working memory load [69].

### Current study

Given reliable associations between variation in emotions and face recognition [44, 46, 48–51, 53], the conceptual overlap of emotions and ER [56, 57], the effects of various ER strategies on memory [62–65], and the associations between memory and face recognition [9, 32, 69–71], it is reasonable to assume that ER strategies might influence face recognition. Surprisingly, however, no previous studies have explicitly investigated this assumption (e.g., for reviews see 1–3]. Therefore, the present study aimed to examine the relationship between individual differences in ER and face recognition. This is the first study to examine how face memory (Experiment 1) and face perception (Experiments 2 and 3) might be affected by a range of adaptive and maladaptive ER strategies.

## Experiment 1

### Method

#### Participants

Forty-two undergraduate students (21 females and 21 males) from Qatar University (Qatar) volunteered to participate in this experiment. Participant’s mean age was 20.1 years (*SD* = 2.1) and all had normal or corrected to normal vision and none had any history of psychopathology using a self-reported item asking about whether they had any current or previous mental health problems. Ethical approval for participation in the three experiments in this study was provided by Qatar University's institutional review board (QU-IRB) and all methods were administered in accordance with QU-IRB guidelines and regulations. Written informed consent was obtained from all participants for being included in the experiments.

#### Measures

*(1) Face immediate memory task*. This task consists of 20 images of target faces along with 40 corresponding 10-face target-present/ target-absent lineups. These stimuli were taken from an Egyptian face database [72]. Target face images were stills captured from a high-quality digital camcorder, whereas all lineup images were photographs taken from a high-quality digital camera. The target and line-up images were taken on the same day under the same lighting conditions, and each image showed a very similar full-face pose of young, clean-shaven Egyptian young men who were undergraduate students in an Egyptian university. Notably, only male faces were used in this study as the vast majority of females in Arab countries are wearing headscarves, which have particular influences on face perception and recognition [16, 73]. All images were shown in grey-scale and the size of each face was approximately 5–7 cm. Full details about the construction of this task can be found in Megreya and Burton (2008) [72].

**Fig 1:**
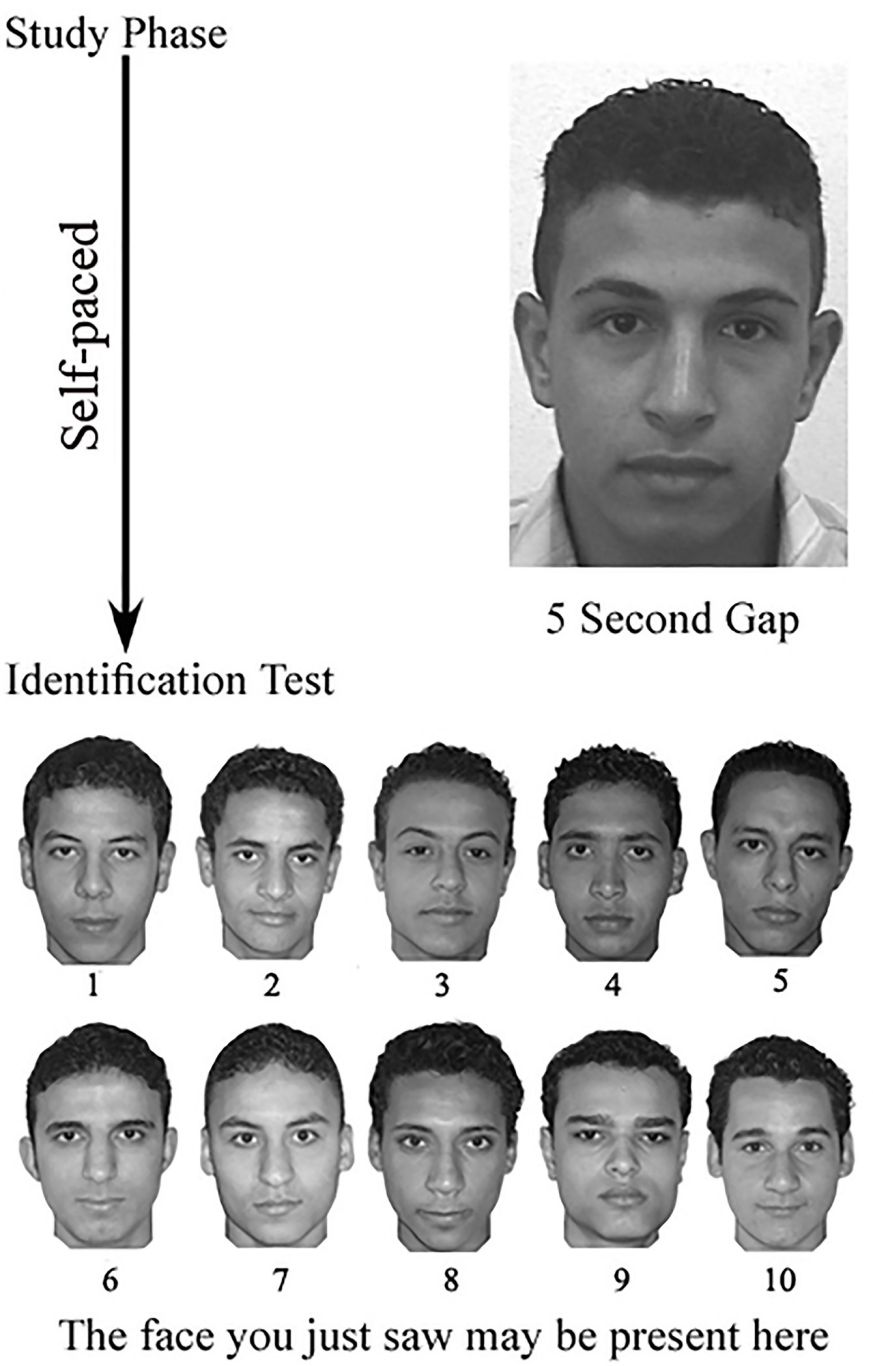
A schematic representation of the unfamiliar immediate memory task.

**Fig 2:**
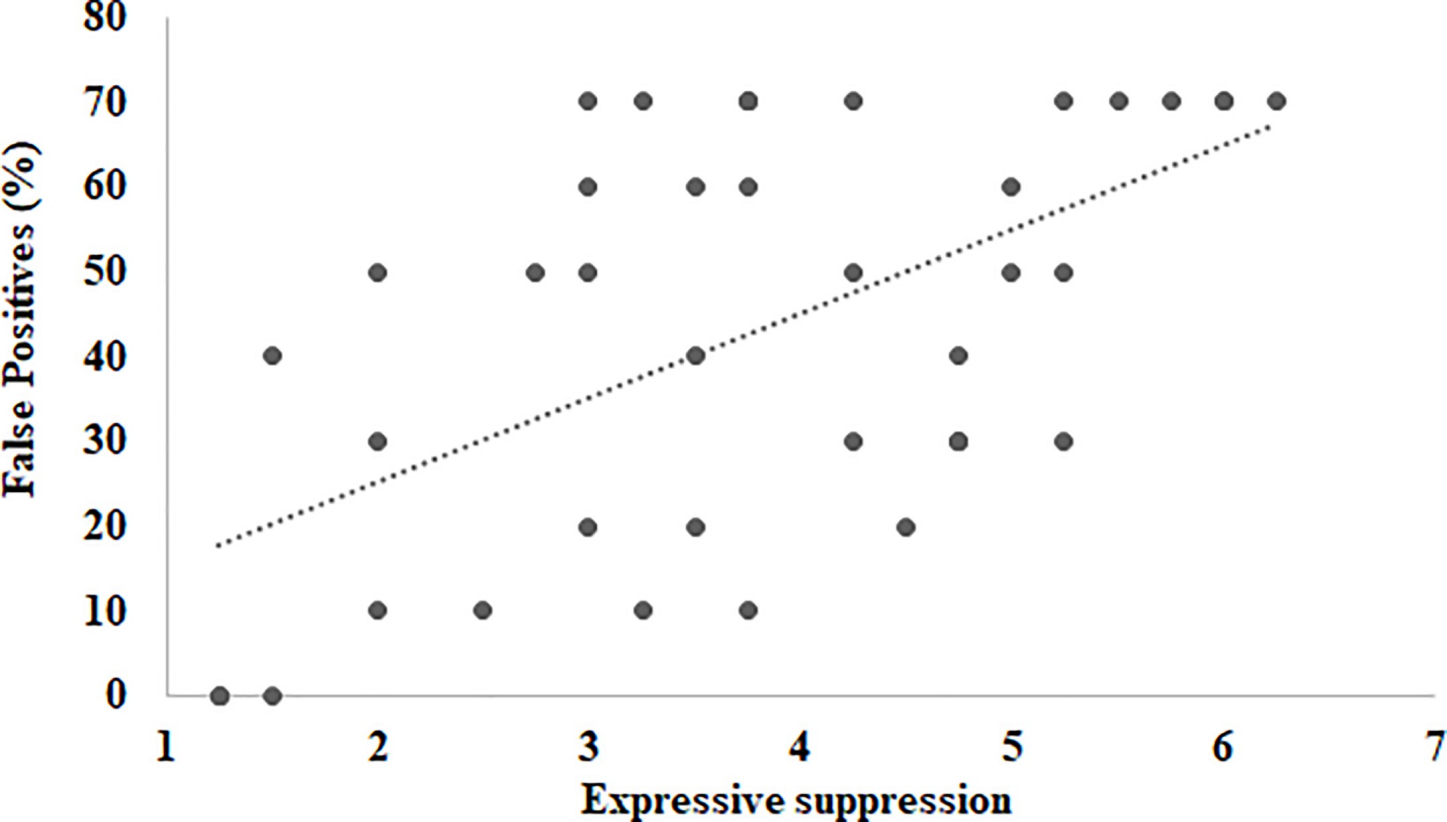
Scatter plots for the relationship between expressive suppression and false positives in face immediate memory in Experiment 1. r (40) = .57, p< 0.001, with 95% confidence internals of .32 to .74.

Participants were tested individually in a session of approximately 10 minutes. On each trial, they were shown (i) a target face; (ii) an intervening interval of 5 seconds; (iii) a 10 face target-present or target-absent lineup. Fig 1 shows a schematic representation of these procedures. The individuals pictured in Figs 1 and 2 have provided written informed consent (as outlined in PLOS consent form) to publish their image alongside the manuscript. There was no time limit for studying the targets. Consistent with previous studies [29, 72], participants were instructed to study each target until they felt confident that they could recognized him in a subsequent 10-face lineup test. After a 5-second gap, participants were instructed that the face they had just seen might or might not be present in the lineup.

Each participant completed 20 trials: 10 target-present and 10 target-absent. The presence of targets was counter-balanced across the experiment so that each target appeared equally often in target-present and target-absent lineups. Using an answer sheet, participants were asked to write down the number of the face in the lineup or mark X if they decided that the target was not present.

*(2) Emotion Regulation Questionnaire (ERQ) [74]*. Gross and John (2003) developed the Emotion Regulation Questionnaire (*ERQ)*, an instrument designed to measure the two most commonly used ER strategies: cognitive reappraisal (antecedent-focused) and expressive suppression (response-focused) [74]. Cognitive reappraisal is defined as “a form of cognitive change that involves construing a potentially emotion-eliciting situation in a way that changes its emotional impact”, whereas expressive suppression is defined as “a form of response modulation that involves inhibiting ongoing emotion-expressive behavior” *[74*, p. 349]. The ERQ is a 10-item self-report measure of cognitive reappraisal (6 items) and expressive suppression (4 items), using a 7-point Likert-type scale ranging from 1 (*strongly disagree*) to 7 (*strongly agree*). Gross and John (2003) reported moderate internal reliabilities for cognitive reappraisal (α = 0.79) and expressive suppression (α = 0.73), with no inter-correlation (r = – 0.01). ERQ has dominated the ER literature and has been translated into many different languages including Arabic [75]. Across four Arab countries (Egypt, Kuwait, Qatar, and Kingdom of Saudi Arabia), Cronbach alpha reliability rates for cognitive reappraisal and expressive suppression were acceptable to good, ranging from 0.75 to 0.85 [75].

### Results

In the face immediate memory task, given previous findings that performances on these trials are dissociable [9, 29, 72], participant’s responses to target-present versus target-absent arrays were assessed separately. In target-present trials, we measured hits (the correct identification of the target face), misses (the incorrect decision that the target was absent), and misidentification (the identification of a distractor face). In target-absent trials, false positives (the incorrect decision that the target was present) were calculated. In addition, we report the overall accuracy by combining hits and correct rejection (the complement of false positives). For the ERQ, we report the averages of individuals’ responses on the items measuring cognitive reappraisal and expressive suppression. Table 1 shows descriptive statistics for these measures.

**Table 1:** Descriptive statistics for participants’ responses on the face immediate memory task (%) and the ERQ.

Measure	M	SD	Minimum	Maximum
*Face Immediate memory*				
Overall accuracy	61.9	16.8	30	100
Hits	66.2	17.2	30	100
Miss	17.4	15.9	0	50
Misidentification	16.9	13.9	0	50
False Positives	42.4	24.5	0	70
*Emotion Regulation Questionnaire*				
cognitive reappraisal	4.9	.9	2.5	6.7
expressive suppression	3.7	1.4	1.3	6.3

Table 2 shows Pearson Correlation coefficients between face immediate memory and ERQ strategies. Cognitive reappraisal did not correlate with any measures of face memory (*mean r* = .06). However, expressive suppression evidenced a moderate to strong negative correlation with the overall accuracy of face immediate memory (*r* = -.52, p < 0.001). Specifically, higher levels of expressive suppression were associated with a lower level of overall face-memory accuracy and higher rates of misidentifications and false positives. Fig 2 shows scatter plots for the relationship between expressive suppression and false positives. No associations were found for hits and misses.

**Table 2:** Correlations between face immediate face memory and ERQ strategies.

	Cognitive reappraisal	Expressive suppression
Overall accuracy	.06	-.52**
Hits	.06	-.20
Miss	-.13	-.14
Misidentification	.10	.40**
False Positives	-.03	.57**

Note

** = p< 0.01.

### Discussion

Consistent with previous experiments [72], overall performance on the face immediate memory task was rather low (62%), but there were wide individual differences ranging from 30% to 100%. Similarly, wide ranges of individual differences in ERQ strategies were evident (see Table 1), replicating previous studies [74]. The overall accuracy of immediate face memory negatively correlated with expressive suppression. This correlation was mainly derived from misidentification and false positive, which are positively correlated with each other [9, 29, 72]. However, there was no correlation between any measure of face memory and cognitive reappraisal.

The negative correlation found between expressive suppression and face immediate memory can be explained by integrating two main findings within the existing literature. First, expressive suppression has been found to associate with negative emotions and stress-related symptoms [74]. For example, Butler et al. (2003) found that expressive suppression disrupted social communication, had a negative impact on the regulators' emotional experience, and increased stress levels as indicated by increased blood pressure [76]. Second, face recognition studies have found that negative emotions, especially anxiety, had detrimental effects on performances on a range of face recognition tasks [44, 46, 48–51, 53]. It is therefore possible that expressive suppression might mediate the negative relationship between emotions and face recognition. Future studies are needed to more explicitly examine this possibility, though.

The negative correlation between expressive suppression and face memory also converges with the results of Richard and Gross (2000) that expressive suppression, but not cognitive reappraisal, is associated with poor memory [62], as previous studies reported positive associations between memory and face recognition [9, 32, 69–71]. Although some studies have found that face memory is positively correlated with face perception [26], face recognition theories suggested a dissociation between face perception and face memory [13]. Therefore, to more clearly explicate this potential dissociation, Experiment 2 aimed to examine the associations between ERQ strategies and a face-matching task.

## Experiment 2

Experiment 1 found detrimental effects of expressive suppression on some aspects of immediate face memory. The aim of the present experiment was to replicate this finding using a range of 1-in-1 face-matching tasks, in which participants were presented with pairs of unfamiliar faces and they were asked to make same/different decisions. Surprisingly, previous studies have repeatedly demonstrated that performance on this seemingly easy task is rather poor, with an error rate of roughly 20 per cent for overall accuracy [9, 72]. In addition, to maximize individual differences, we involved highly challenging face-matching conditions including same- vs. other-race face images taken in the same day or different times. Previous studies have reported that other-race faces are perceived and recognized less accurately than own-race faces [15–18] and that matching performance highly degrades when the task involves images taken months apart [77].

### Method

#### Participants

Fifty-three undergraduate students (28 females and 25 males) from Qatar University volunteered to participate in this experiment. The participants’ mean age was 19.5 years (*SD* = 1.6) and all had normal or corrected to normal vision. None had any history of psychopathology as self-reported and none had participated in Experiment 1.

#### Measures

**Fig 3:**
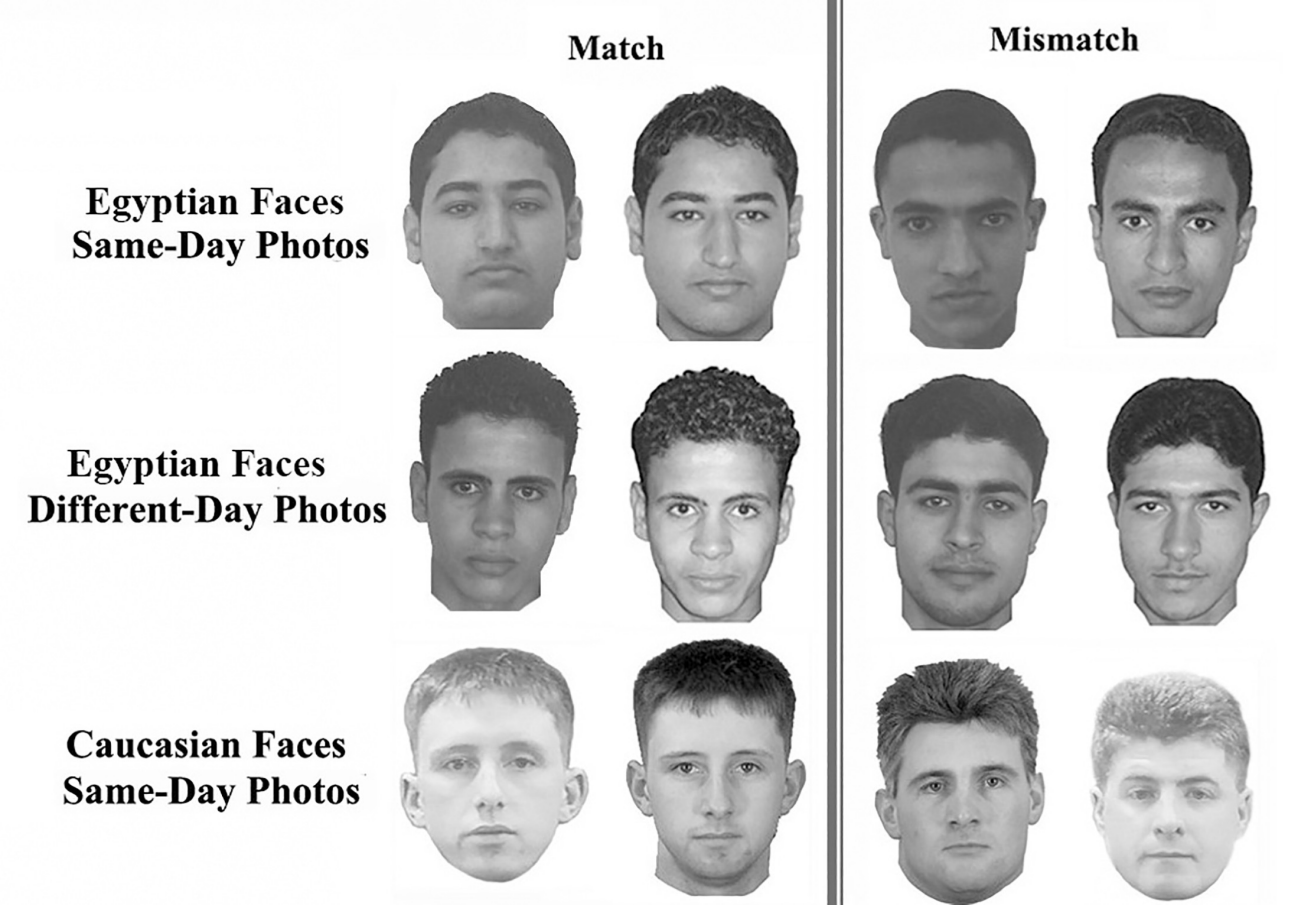
Examples of face-matching tasks used in Experiment 2 and 3. Regarding copyright issues, we could not present examples for the UK face images that were taken in different times.

*(1) Face-matching tasks*. A total of 200 match/mismatch pairs of Egyptian and UK male unfamiliar faces were used in this experiment. For each face nationality, there were 60 same-day photo pairs (30 matches and 30 mismatches) and 40 different-day photo pairs (20 matches and 20 mismatches). Fig 3 shows examples of these stimuli.

The Egyptian and UK same-day face-matching pairs were taken from Megreya and Burton (2008 and 2006), respectively [9, 72]. All images showed a full-face view with a neutral facial expression. On the one hand, each matching pair consisted of a still photograph of a target face captured by a high-quality video camera and a photograph depicting the same person that was taken from a high-quality digital camera in the same day and under the same lighting conditions. On the other hand, each mismatching pair consisted of a still photograph of a target face and a digital photograph depicting a distractor face.

The Egyptian and UK different-day face-matching pairs were taken from Megreya et al., (2013) and White, Kemp, Jenkins, and Burton, (2014), respectively [77, 78]. For the Egyptian stimuli, each matching pair consisted of a still photograph of a target and a photograph depicting the same face that was taken months apart, with an average of roughly seven months, whereas mismatch pairs consisted of stills and photographs depicting different faces. Like the same-day stimuli, all Egyptian different-day images showed a full-face view with a neutral facial expression. In addition, all stills and photographs were taken from the same camcorder and digital cameras as used in the same-day condition and under the same lighting conditions. The UK face pair stimuli were constructed using sets of images depicting Australian and British celebrities taken from the internet. Matching pairs consisted to two images depicting the same face that were taken during different times and showed different expressions, whereas mismatching pairs consisted of two images of two different people. Notably, all celebrities were unknown to the participants in this study as confirmed by all of them after the experiment.

Each participant completed 100 trials (15 Egyptian same-day matches; 15 Egyptian same-day mismatches; 10 Egyptian different-day matches; 10 Egyptian different-day mismatches; 15 UK same-day matches; 15 UK same-day mismatches; 10 UK different-day matches; 10 UK different-day mismatches). Two versions of stimuli were created to counter-balance match/mismatch trials so that each target face was equally presented in match and mismatch pairs across the experiment.

*(2) Emotion Regulation Questionnaire (ERQ) [74].* This was the same instrument described in Experiment 1 above.

### Results

Three indices were calculated for the face-matching tasks. These included (i) hits (correct decision that the two faces in matching pairs depict the same identity), (ii) false positives (FPs; false decision that the two faces in mismatching depict the same identity), and (iii) overall accuracy (hits plus correct rejections; the complement of FPs). Consistent with Experiment 1, we report the averages of individuals’ responses on the ERQ items measuring cognitive reappraisal and expressive suppression. Table 3 shows descriptive statistics for these measures.

**Table 3:** Descriptive statistics for participants’ responses on the 1-in-1 face-matching tasks and ERQ strategies in Experiment 2.

	M	SD	Minimum	Maximum
*General Performance*				
Overall Accuracy	79.8	8.5	51.7	92.9
Hits	81	11.4	50	96.7
FPs	21.5	14.5	1.7	63.3
*Performance on Egyptian same-day task*				
Overall Accuracy	85.4	10.8	46.7	100
Hits	91.1	11.9	40	100
FPs	20.4	17.8	0	80
*Performance on Egyptian different-day task*				
Overall Accuracy	81.5	10.4	55	100
Hits	75.6	17.8	20	100
FPs	12.5	13.8	0	50
*Performance on UK same-day task*				
Overall Accuracy	81	11.4	50	100
Hits	84.4	11.2	60	100
FPs	22.3	19.2	0	73.3
*Performance on UK different-day task*				
Overall Accuracy	71.1	10.0	50	90
Hits	73.1	19.6	20	100
FPs	31.0	20.1	0	80
*ERQ*				
Reappraisal	5.3	0.9	3.2	6.8
Suppression	3.7	1.5	1	6.3

#### Correlations between face matching and ERQ strategies

**Fig 4:**
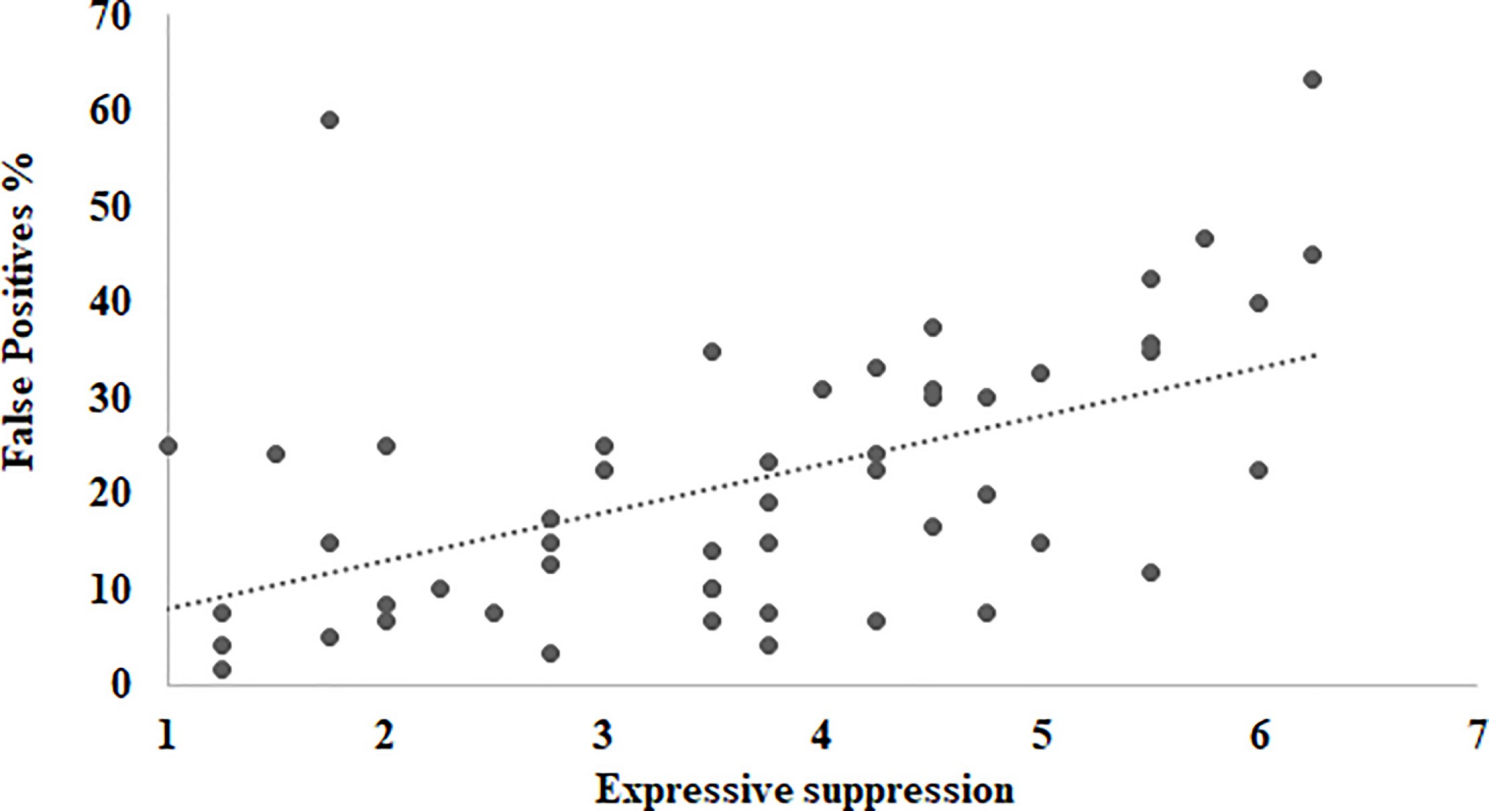
Scatter plots for the relationship between expressive suppression false positives in the overall face-matching task in Experiment 2. r (50) = .51, p< 0.001, with 95% confidence internals of .22 to .75.

Table 4 shows Pearson correlation coefficients between participants’ performances on the face matching tasks and ERQ strategies. For general performance across the four face-matching tasks, there were no significant correlations between all matching measures and cognitive reappraisal (*mean r* = -.01). However, expressive suppression correlated negatively with the overall accuracy (*r* = -.34, p = 0.01) and positively with FPs (*r* = .51, p < 0.001). Therefore, higher levels of expressive suppression were associated with a lower level of overall face-matching performance and higher rates of false positives. Fig 4 shows scatter plots for the relationship between expressive suppression and false positives in the overall face-matching task. Moderate-to-strong positive correlations between expressive suppression and FPs were consistently found across the four face-matching tasks (*mean r* = .39). However, no correlation was found between expressive suppression and hits (see Table 4).

**Table 4:** Correlations between face matching and ERQ strategies in Experiment 2.

	Reappraisal	Suppression
General performance		
Overall Accuracy	-0.01	-.34**
Hits	0.06	0.14
FPs	0.06	0.51**
Performance on Egyptian same-day task		
Overall Accuracy	0.05	-0.42**
Hits	0.15	-0.03
FPs	0.04	0.48**
Performance on Egyptian different-day task		
Overall Accuracy	0.17	-0.11
Hits	0.18	0.18
FPs	-0.02	0.39*
Performance on UK same-day task		
Overall Accuracy	-0.04	-0.31*
Hits	0.08	0.02
FPs	0.09	0.38*
Performance on UK different-day task		
Overall Accuracy	-0.23	-0.23
Hits	-0.16	0.19
FPs	0.07	0.40*

Note

* = p < 0.05

** = p< 0.01.

#### Correlations among the four face-matching tasks

Table 5 shows Pearson correlation coefficients among the four face-matching tasks. There were strong positive correlations among all tasks with the following exceptions. Hit scores in matching Egyptian faces that were taken during the same time did not correlate with hits of matching UK faces that were taken during different times. In addition, no correlation was observed for FPs in matching Egyptian and UK faces in the different time condition.

**Table 5:** Correlations among the four face-matching tasks in Experiment 2.

	Egyptian/ Same Time	Egyptian/ Different Times	UK/ Same Time
*Overall Accuracy*			
Egyptian/ Different Times	.48**		
UK/ Same Time	.79**	.39**	
UK/ Different Times	.41**	.45**	.49**
*Hits*			
Egyptian/ Different Times	.48**		
UK/ Same Time	.48**	.52**	
UK/ Different Times	.19	.35*	.57**
*False Positives*			
Egyptian/ Different Times	.72**		
UK/ Same Time	.80**	.57**	
UK/ Different Times	.42**	.27	.51**

Note

* = p < 0.05

** = p< 0.01.

#### Performances across the four face-matching tasks

The comparability of the Egyptian and UK same day face-matching tasks were higher than that of the different day tasks. On the one hand, the Egyptian different day task consisted of images of targets that were taken several months apart using the same cameras and under the same lighting conditions, similar to the Egyptian same day task. On the other hand, the UK different day task involved ambient images taken from the internet. With this caution, we examined the differences among participants’ performances on these four face-matching tasks. Matching measures were subjected to a series of 2 (face nationality: Egyptian vs. UK) x 2 (times: same-day vs. different-day photos) within-participant Analysis of Variances (ANOVAs). For overall accuracy, there were main effects of face nationality, *F* (1,51) = 55.98, p < 0.001, showing that Egyptian faces were matched more accurately than UK ones (83.5% vs. 76%), and image times, *F* (1,51) = 29.06, p<0.001, indicating that same-day faces were matched more accurately than different-day ones (83.2% vs. 76.3%). In addition, there was an interaction between these two factors, *F* (1,51) = 15.51, p<0.001. Subsequent Simple Main Effects (SMEs) reported other-race effects using both same-day and different-day stimuli, *F*s (1,51) = 9.66 and 55.84, ps ≤ 0.01, and confirmed the image time effects using both same-race (Egyptian) and different-race (UK) faces, *F*s (1,51) = 4.51 and 30.25, ps ≤ 0.05. Consistently, significant main effects of face nationality were noticed for both hits and false positives, *F*s (1,51) = 5.96 and 29.63, ps ≤ 0.05. Image times had significant main effect on hits, *F* (1,51) = 63.16, p < 0.001, but not for false positives, *F* < 1.

### Discussion

This experiment examined the correlation between participant performance on a range of face-matching tasks, with different levels of difficulty, and ERQ strategies. Consistent with previous studies [9, 72], the task of matching images depicting same-race unfamiliar faces that were taken on the same day was relatively error-prone so that participants falsely rejected roughly 10% of matching pairs and falsely accepted roughly 20% of mismatching pairs. In addition, consistent with Megreya et al. (2013), a lower level of performance was noticed when participants had to match same-race face images that were taken months apart as they falsely rejected roughly 25% in matching pairs [77]. Furthermore, the present results replicated the well-established other-race effect [18] in that participants matched their own-race faces more accurately than other-race faces when the images were taken in the same day (85% vs. 81%) or in different times (81% vs. 71%).

Experiment 1 found that performance on a face immediate memory task did not correlate with cognitive reappraisal, whereas overall accuracy, misidentifications and false positives were associated with a high use of expressive suppression. Providing converging evidence in support of the stability of these findings, using a range of perceptual tasks, the present experiment demonstrated a close to zero correlation between face-matching performances and cognitive reappraisal, while a lower level of overall accuracy and higher rates of false positives were associated with a higher use of expressive suppression. Therefore, maladaptive ER strategies, in general, might have detrimental effects on face recognition. To examine this suggestion further, Experiment 3 investigated associations between the same four face-matching tasks and a broader range of adaptive versus maladaptive cognitive ER strategies.

## Experiment 3

Experiments 1 and 2 both found that expressive suppression was negatively associated with the accuracy of face memory and face perception, respectively. This ER strategy has been generally considered as maladaptive in that it is associated with increased rates of negative emotions and psychopathology [61]. The ER literature, however, suggests that additional maladaptive ER strategies exist which may similarly be associated with face-perception abilities. For example, Garnefski et al., (2001) developed a nine-factor framework for the cognitive emotion regulation strategies thought to encompass the various ways in which people cognitively manage and control their emotions during or after the experience of a stressful event [60]. These factors include self-blame, acceptance, rumination, positive refocusing, refocus on planning, positive reappraisal, putting into perspective, catastrophizing, and other-blame. Garnefski et al. (2001) found that these nine factors could be classified into two boarder factors: adaptive strategies (which include Positive refocusing, Positive reappraisal, Putting into perspective, Refocus on planning and Acceptance) and maladaptive strategies (which include Rumination, Self-blame, Other-blame and Catastrophizing) [60]. Megreya, Latzman, Al-Attiyah, & Alrashidi, 2016) replicated the nine-factor structure of the CERQ among four Arab countries (Egypt, Kingdom of Saudi Arabic, Kuwait, and Qatar) [79]. To further examine how adaptive versus maladaptive emotion regulation strategies may influence face recognition, Experiment 3 was conducted to investigate individual differences in across these cognitive ER strategies and face perception.

### Method

#### Participants

Fifty-two graduate and undergraduate students from Qatar University (28 females and 24 males) volunteered to participate in this experiment. Their mean age was 26.4 years (*SD* = 4.1) and all had normal or corrected to normal vision. None had history of psychopathology as self-reported and none had participated in Experiments 1 and 2.

#### Measures

*(1) Face-matching tasks*. This experiment used the same face-matching tasks that were used in Experiment 2. These required participants to match sets of match/ mismatch pairs of unfamiliar faces, which belonged to the same- or different-race and taken on the same day or during different times (see Fig 3 for examples).

*(2) Cognitive Emotion Regulation Questionnaire (CERQ)* [60]. The Cognitive Emotion Regulation Questionnaire (CERQ) assesses a variety of cognitive strategies that people tend to use following the experience of negative events and situations. The CERQ measures nine cognitive ER strategies, which were defined as following [80]:

- *Self-blame*: the thoughts of blaming oneself for what she/he has experienced,
- *Acceptance*: the thoughts of resigning what has happened,
- *Rumination*: thinking all the time on the feelings and thoughts associated with negative events,
- *Positive refocusing*: thinking of other, pleasant matters instead of the actual event,
- *Refocus on planning*: thinking on potential steps to deal with negative events,
- *Positive reappraisal*: thinking of attaching a positive meaning to the event in terms of personal growth,
- *Putting into perspective*: the thoughts of playing down the seriousness of a negative event as compared to other events,
- *Catastrophizing*: the explicit emphasize of the terror of negative events,
- *Other-blame*: the thoughts of putting the blame for what one has experienced on others.

The CERQ is a 36-item self-report measure of these nine cognitive ER strategies that individuals tend to use after experiencing a stressful life event. It consists of nine subscales, which measure a range of adaptive (Positive refocusing, Refocus on planning, Positive reappraisal, and Putting into perspective) and maladaptive (Self-blame, Acceptance, Rumination, Catastrophizing, and Other-blame) cognitive ER strategies. Each subscale consists of 4 items, using a 5-point Likert-type scale ranging from 1 (*almost never*) to 5 (*almost always*). Therefore, the scores of each subscale range between 4 and 20, and a high score reflects a greater use of the CERQ strategy. All of the nine-CERQ factors positively correlated with each other (*r*s ranged between .20 and .62), with moderate to high Cronbach’s alpha reliabilities (αs ranged between 0.68 and 0.83) and high test-retest stability (*r*s ranged between 0.41 and 0.59). The Arabic version of the CERQ has similar adequate psychometric properties in a range of Arabic countries, including Qatar [79, 80]. Specifically, Megreya et al (2016) replicated the nine-factor structure (CERQ strategies) and the higher two-factor solution (adaptive versus maladaptive strategies) of the Arabic version of the CERQ, with acceptable to good Cronbach reliability rates for the nine CERQ subscales (which ranged from 0.67 to 0.86) [79].

### Results

#### Correlations between face matching and CERQ strategies

**Fig 5:**
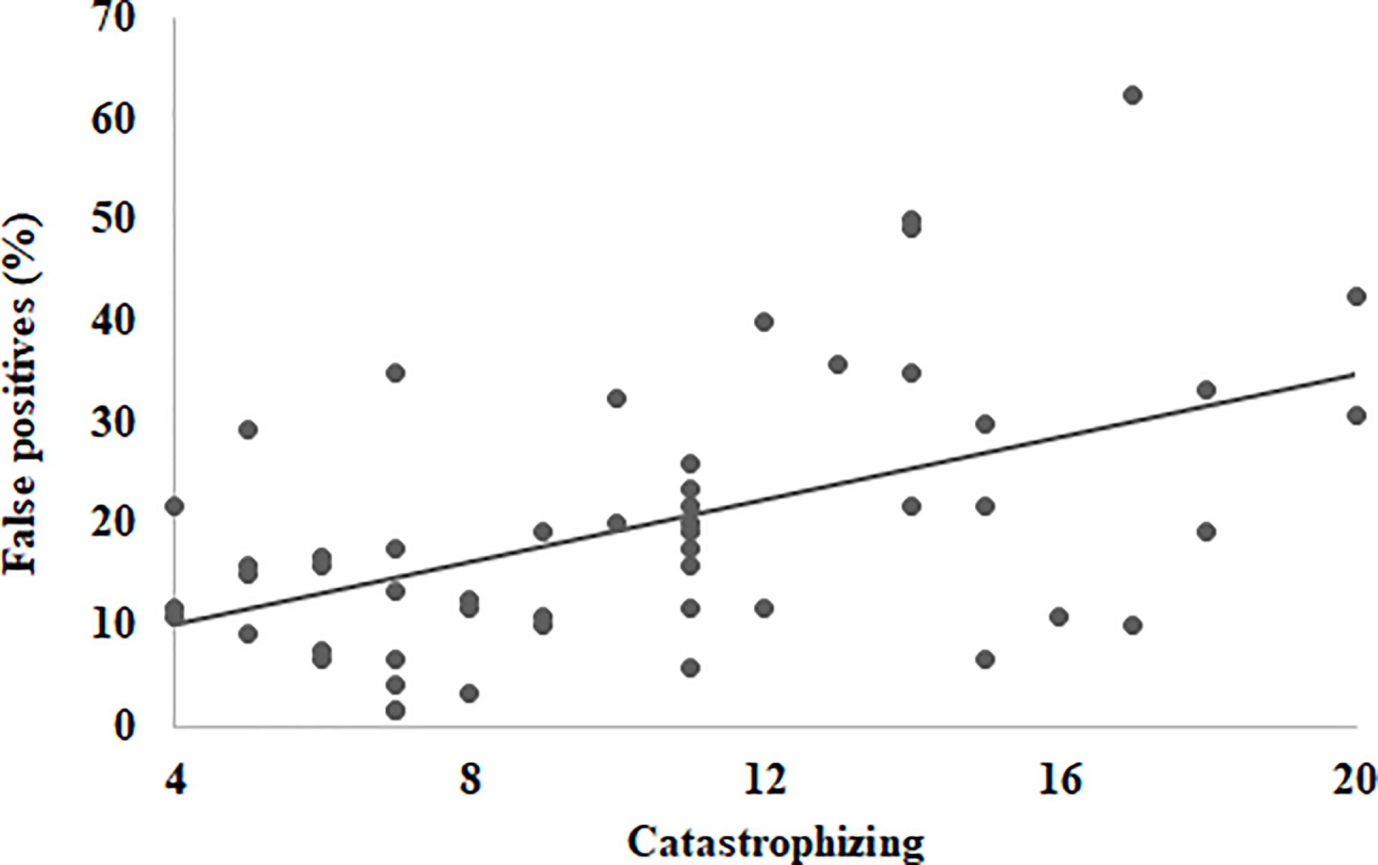
Scatter plots for the relationship between catastrophizing and false positives in the overall face-matching task in Experiment 3. r (50) = .51, p< 0.001, with 95% confidence internals of .29 to .68.

Table 6 shows descriptive statistics for participants’ responses on the face-matching tasks and the CERQ. Table 7 shows Pearson correlation coefficients between participants’ performances on the face matching tasks and CERQ strategies. For participants’ general performance in the four face-matching tasks, overall face-matching accuracy correlated negatively with self-blame, rumination, and catastrophizing. Hits correlated negatively with self-blame (r = -.32, p = 0.02 and rumination (r = -.28, p = 0.04) whereas false positives correlated positively with catastrophizing (r = .51, p < 0.001). Fig 5 shows scatter plots for the relationship between catastrophizing and false positives in the overall face-matching task. Across all of the four face-matching tasks, strong positive correlations between false positives and catastrophizing were consistently reported (*r*s ranged from .35 to .45, *p*s < 0.01, *mean r* = .40). However, no correlation was found between the face matching measures and the other eight CERQ factors, especially for matching own-race (Egyptian) faces. When broad dimensions were considered, no correlation was found between adaptive strategies and any of face-matching measures across all tasks. Importantly however, maladaptive strategies correlated negatively with overall accuracies in all face-matching tasks (rs ranged from -.28 to -.45, *p*s < 0.05, mean r = -.35) but not in the task of matching Egyptian different-day faces (r = -.15, p = .27). That is, maladaptive cognitive emotion regulation strategies, especially catastrophizing, were associated with lower levels of overall face-matching performances and higher rates of false positives.

**Table 6:** Descriptive statistics for participants’ responses on the face matching tasks and CERQ in Experiment 3.

	M	SD	Minimum	Maximum
*Face matching tasks*				
*General Performance*				
Overall Accuracy	80.2	7.8	59.2	93.8
Hits	80.2	12.3	50.0	100
FPs	19.8	13.3	1.7	62.5
*Performance on Egyptian same-day task*				
Overall Accuracy	85.8	8.5	63.3	100
Hits	90.5	10.1	60	100
FPs	18.8	14.6	0	60
*Performance on Egyptian different-day task*				
Overall Accuracy	81.2	11.4	55	100
Hits	76.2	20.3	20	100
FPs	13.8	13.6	0	60
*Performance on UK same-day task*				
Overall Accuracy	82.0	9.8	56.7	100
Hits	83.7	14.1	40	100
FPs	19.7	18.0	0	86.7
*Performance on UK different-day task*				
Overall Accuracy	71.8	10.8	45	95
Hits	70.6	17.6	20	100
FPs	26.9	21.3	0	80
*CERQ*				
Self-Blame	12.3	3.5	5	19
Acceptance	13	2.8	4	20
Rumination	14.6	3.9	5	20
Positive Refocusing	13.3	4.0	5	19
Refocus on Planning	16.1	3.2	6	20
Positive Reappraisal	15.8	3.3	5	20
Putting into Perspective	15.0	3.5	4	20
Catastrophizing	10.4	4.4	4	20
Other-blame	10	3.4	4	19
Adaptive strategies	60.3	11.7	20	77
Maladaptive strategies	60.3	13.8	22	91

**Table 7:** Correlations between performances on the face-matching tasks and CERQ strategies in Experiment 3.

	Self	Acc	Rum	PRef	Plan	PosR	Pers	Cat	Other	Adaptive	Maladaptive
*General Performance*											
Overall Accuracy	-.36**	-.20	-.32*	-.14	.08	-.09	-.01	-.42**	-.12	-.05	-.38**
Hits	-.32*	-.10	-.28*	-.08	-.15	-.14	.08	.02	-.01	-.08	-.17
FPs	.12	.15	.11	.09	-.23	-.02	.08	.51**	.13	-.02	.29*
*Performance on Egyptian same-day task*											
Overall Accuracy	-.21	-.19	-.22	-.14	.03	-.09	-.01	-.36**	-.03	-.10	-.28*
Hits	-.27	-.12	-.18	-.16	-.19	-.05	.08	.04	.16	-.18	-.09
FPs	.06	.14	.14	.06	-.17	.06	.08	.45**	.15	-.01	.26
*Performance on Egyptian different-day task*											
Overall Accuracy	-.17	-.19	-.17	-.05	.03	-.12	.15	-.17	.13	-.01	-.15
Hits	-.15	-.05	-.19	-.09	-.20	-.23	.06	.09	.17	-.14	-.03
FPs	.06	.24	.01	-.05	-.34	-.15	-.16	.43**	.04	-.20	.21
*Performance on UK same-day task*											
Overall Accuracy	-.32*	-.26	-.33*	-.13	.10	-.04	-.11	-.51**	-.24	-.06	-.45**
Hits	-.34**	-.13	-.34*	-.18	-.12	-.17	-.03	-.27	-.18	-.15	-.36**
FPs	-.01	.18	.09	-.01	-.20	-.08	.10	.35*	.12	-.05	.20
*Performance on UK different-day task*											
Overall Accuracy	-.38**	-.01	-.25	-.11	.09	.01	-.01	-.28*	-.23	-.01	-.31*
Hits	-.20	-.02	-.18	.12	.03	.13	.20	.14	-.16	.15	-.10
FPs	.22	.01	.10	.21	-.07	.10	.18	.39**	.10	.13	.23

*Note*: Self = Self-blame; Acc = Acceptance; Rum = Rumination; PRef = Positive Refocusing; Plan = Refocus on Planning; PosR = Positive Reappraisal; Pers = Putting into Perspective; Cat = Catastrophizing; Other = Other-blame; FPs = False positives

* = p<0.05

** = p<0.01.

#### Correlations among the four face-matching tasks

Table 8 shows Pearson correlation coefficients among the four face-matching tasks. There were strong positive correlations among all tasks (*r*s ranged from .31 to .67, mean *r* = .49).

**Table 8:** Correlations among the four face-matching tasks in Experiment 3.

	Egyptian/ Same Time	Egyptian/ Different Times	UK/Same Time
*Overall Accuracy*			
Egyptian/ Different Times	.50**		
UK/ Same Time	.67**	.43**	
UK/ Different Times	.51**	.31*	.36**
*Hits*			
Egyptian/ Different Times	.61**		
UK/ Same Time	.66**	.61**	
UK/ Different Times	.32*	.37*	.44**
*False Positives*			
Egyptian/ Different Times	.53**		
UK/ Same Time	.60**	.48**	
UK/ Different Times	.50**	.36**	.49**

Note

* = p < 0.05

** = p< 0.01.

#### Performances across the four face-matching tasks

A series of 2 (face nationalities) x 2 (image times) within-participants ANOVAs were conducted using the three face-matching measures. Face nationality and image time factors yielded significant main effects on overall accuracy, *F*s (1,51) = 43.14 and 47.49, *p*s < 0.001, and hits, *F*s (1,51) = 13.83 and 71.24, *p*s < 0.001, showing same-race and same-day advantages; respectively. For false positives, the same-race advantage was also noticed, *F* (1,51) = 14.68, *p* < 0.001, but there was no main effect of image time, F (1,51) < 1. Face nationality and image time yielded interactions for overall accuracy, *F* (1,51) = 6.43, *p* = 0.01, and false positives, *F* (1,51) = 12.56, *p* < 0.001, but not for hits, F(1, 51) <1, p = .71. The results of Simple Main Effects of overall accuracy and false positives can be found as a supplementary file.

### Discussion

Replicating results of Megreya et al (2013) [77] and Experiment 2, hit rates dropped from 90.5% to 76.1% when observers were asked to match same-race face images that were taken on the same day or months apart. In addition, consistent with Experiment 2, hit rates dropped from 83.7% to 70.6% when they had to match other-race face images that were taken on the same day or during different times. Indeed, a large body of experimental studies have provided good evidence that face matching is rather error-prone [9, 72]. However, the vast majority of these studies have used face-matching stimuli that were photographed on the same day and under the same lighting conditions. Consistent with a previous suggestion [77], the present results suggest that these previous experimental studies likely provide an underestimate of the challenges of face identity verification in security settings such as country borders where passport officers have to match face identities of different ethnic groups to their passport photographs that would not be never taken on the same day.

The results of this experiment also improve our understanding of the relationship between adaptive versus maladaptive ER strategies and face perception. Adaptive cognitive ER strategies–individually or combined–did not correlate with any face matching measures consistently in all face-matching tasks. This finding converges with the results of Experiments 1 and 2 that cognitive reappraisal–as an adaptive ER strategy–does not correlate with face memory and face perception. Importantly, however, higher use of combined maladaptive cognitive ER strategies, as well as self-blame, rumination, and catastrophizing more specifically, were associated with lower levels of overall accuracy and higher rates of false positives in the overall face-matching task. Within each face-matching task, there were inconsistent correlations with self-blame and rumination, but catastrophizing was positively correlated with false positives. The positive correlations between catastrophizing and false positives were strong in magnitude (with mean *r* = .49) and robust across all of the four face-matching tasks (see Table 7).

## General discussion

Across 3 experiments, the current research examined for the first time associations between individual differences in a range of adaptive versus maladaptive ER strategies and face recognition. Using an immediate face-memory paradigm, in which observers had to identify a self-paced learned unfamiliar face from a 10-face target-present/ target-absent line-up, Experiment 1 reported that higher levels of expressive suppression, but not cognitive reappraisal, were associated with a lower level of overall face-memory accuracy and higher rates of misidentifications and false positives. Experiment 2 replicated these findings using a range of face-matching tasks, where observers were asked to match pairs of same-race or different-race face images taken on the same day or during different times. Higher levels of expressive suppression were associated with a lower level of overall face-matching performance and higher rates of false positives, but cognitive reappraisal did not correlate with any face-matching measure. Experiment 3 revealed that the higher use of maladaptive cognitive ER strategies, especially catastrophizing, was associated with lower levels of overall face-matching performances and higher rates of false positives. Therefore, the higher use of maladaptive ER strategies in general, and expressive suppression and catastrophizing more specifically, has detrimental effects on face memory and face perception.

Expressive suppression refers to is a form of response modulation that involves inhibiting ongoing emotion-expressive behavior [74], whereas Catastrophizing refers to recurring thoughts about how terrible the event has been and about what one has gone through being the worst thing to happen to a person [80]. Expression suppression is associated with a range of negative social (e.g., disrupted communication) [76] and cognitive (worsened memory) [62] consequences. In addition, evidence indicates social anxiety disorder is associated with an overreliance on expressive suppression [81]. Importantly, results of the current research demonstrated that relatively higher levels of expression suppression have a detrimental effect on face recognition, a social cognitive ability. Catastrophizing is one of most related ER strategies to anxiety disorders generally [82] and social anxiety disorder specifically as socially anxious adults tend to interpret ambiguous events in a negative way and appraise mildly negative events catastrophically [83, 84]. Taken together, given that trait anxiety [48–51] and social anxiety [53] are associated negatively with face recognition, expressive suppression and catastrophizing serve as mediators in the negative relationship between anxiety disorders and face recognition.

As this study is exploratory, the mechanisms by which expressive suppression and catastrophizing affect face recognition are not clear. Notably, these two maladaptive ER strategies were associated with higher false positives but they did not correlate with hits. Intuitively, these findings suggest that the influences of expressive suppression and catastrophizing might be related to processing new faces, rather than old faces. In line with this suggestion, Experiment 1 found that higher rates of misidentifications in an immediate face-memory paradigm were also associated with higher expressive suppression (see Table 2). Nevertheless, future studies are encouraged to explore these possibilities.

It is well-known that emotions interact with many aspects of cognitive processing [85]. For example, Blair et al (2007) found that emotional distractors disrupted goal-directed processing and goal-directed processing disrupted the neurophysiological responses to emotional photographs [86]. Previous studies reported that higher levels of expressive suppression, but not cognitive reappraisal, are associated with lower levels of general memory performance [62]. These findings resulted in a conclusion that “keeping a still face and stiff upper lip decreases one's memory for the details of the unfolding emotion-eliciting situation, whereas cognitively transforming the situation by changing one's thinking does not appear to exact such a cognitive cost” [62, p. 423]. Our results are consistent with these conclusions, in that we found expressive suppression and catastrophizing to be associated with mistaken face identification, especially in target-absent trials. These results have potentially important applications. Indeed, whereas most previous studies that have aimed to improve face recognition have focused on the cognitive representations of faces [17, 78, 87, 88], our findings suggest that improving face recognition ability likely requires a more thorough training program that likely includes a focus on cognitive processing of faces as well as strategies for coping with emotions.

A large body of laboratory studies reported that matching face identities using photographs is highly error-prone, suggesting challenges to real-life security settings (such as airports) in where persons’ identities are verified by matching their faces to photo-IDs [9, 72]. Importantly, however, there is a critical mismatch between face matching in those laboratory studies and realistic security settings. Specifically, the vast majority of studies have used face-matching images that were taken on the same day, while the general appearance of faces does change, even day to day, in realistic settings. Further, people can use their passports as long as they are valid, often for many years. Therefore, in one notable study, Megreya et al (2013) simulated face matching procedures in realistic settings by asking participants to match images of unfamiliar faces that were taken on the same day or several months apart [77]. When face images were taken on the same day, Megreya et al. (2013) reported hit rates of 79% and 90% using the 1-in-10 and 1-in-1 face-matching tasks (respectively) [77]. However, hit rates dropped to 58% and 70% on these tasks when face images were taken several months apart. Consistently, the results of Experiments 2 and 3 showed that hit rates were dropped from 91.2% to only 76.7% when face images were taken on the same day or several months apart.

Nevertheless, these face-matching conditions, matching two images taken in different times that depict a face belonging to the same race, still do not typically match realistic security settings, which involve both own-race and different-race faces. It has been known for many years that matching and recognizing other-race faces are more difficult than matching and recognizing own-race faces [15–18]. Therefore, there are three main challenges for matching faces to photo-IDs in realistic settings. These are (i) matching faces using photographs that is a highly-error prone task [9, 72, 77]; (ii) matching face images that were taken on different times [Experiments, 2 & 3, 77]; and (iii) matching faces that belong to other races [5–18]. The present study compared for the first time between the accuracy of matching other-race faces that were taken on the same day or during different times. Specifically, the results of Experiment 2 and 3 reported that hit rates were dropped from 85.6% to only 73.1% when the face images of other-race faces were taken on the same day or during different times. Therefore, along with our previous study [77], the present results suggests that previous laboratory studies on face matching underestimate its difficulty in real-world situations. Photographs of unfamiliar faces seem to be unreliable proofs of identity, especially if the ID documents do not use very recent images of the holders and if the faces to be matched belong to a different race.

## Limitations and conclusions

The current research is not without limitations. For example, the results showed that a behavioral (expressive suppression) and a cognitive (catastrophizing) emotion regulation strategy–as measured by the ERQ and CERQ (respectively)—correlated negatively with the accuracy of unfamiliar face recognition. Although the ERQ and CERQ are widely-used measures of emotion regulation, these findings need replication using, for example, a behavior measure of expressive suppression [62] and other report-based scales that focus explicitly on behavioral strategies [89]. In addition, as both expressive suppression and catastrophizing are considered maladaptive emotion regulation strategies, it is also important to investigate emotion regulation difficulties [90] influence face recognition. An additional limitation is related to the discrepancy with the way in which the face-matching tasks were set up in this study (where 50% of trails were mismatches) and the identity verification in real-life security settings (where mismatches are far less frequent). In addition, only male faces were used. Finally, it is important to note that the UK different-day face-matching task included ambient images depicting Western celebrities who were definitely unknown to the participants. These images, therefore, likely have different characteristics as compared to the images used in the other face-matching tasks. However, our main interest in this study was not to examine this peculiar effect on the other-race effect.

Limitations notwithstanding, the current research reports for the first time that certain emotion regulation strategies (expressive suppression and catastrophizing) negatively affect the accuracy of face recognition. Although additional research with larger samples is needed to confirm the replicability of these findings, the current research provides new evidence for the association between emotion and cognition [85]. In addition, this study provides a more realistic face-matching procedure suggesting that previous laboratory studies may have underestimated the difficulty of unfamiliar face matching in real-world situations.

## Supporting information

S1 Data The experiments reported in this study. (XLSX)

S1 File Supplementary results for experiments 2 and 3 in this study. (DOCX)

## References

[1] Lander K, Bruce V & Bindemann M (2018). Use-inspired basic research on individual differences in face identification: Implications for criminal investigation and security. Cogn Research: 3, 26 10.1186/s41235-018-0115-6 29984301 PMC6021459

[2] Wilmer JB (2017). Individual differences in face recognition: a decade of discovery. Curr Dir Psychol Sci: 26, 225–230. 10.1177/0963721417710693

[3] Yovel G, Wilmer J B & Duchaine B (2014). What can individual differences reveal about face processing? Front Hum Neurosci: 8, 562 10.3389/fnhum.2014.00562 25191241 PMC4137541

[4] Kress T & Daum I (2003). Developmental prosopagnosia: a review. Behav Neurol: 14, 109–21. 10.1155/2003/520476 14757987 PMC5497561

[5] Susilo T., & Duchaine B. (2013). Advances in developmental prosopagnosia research. Curr Opin Neurobiol: 23, 423–9. 10.1016/j.conb.2012.12.011 23391526

[6] Noyes E, Phillips PJ & O’Toole AJ (2017). What is a super-recogniser? In Bindemann M & Megreya AM (Eds.), *Face processing*: *Systems*, *disorders and cultural differences* (pp. 173–201). NewYork: Nova Science Publishers.

[7] Ramon M, Bobak AK, & White D (2019). Super‐recognizers: From the lab to the world and back again. Br J Psychol: 110, 461–479. 10.1111/bjop.12368 30893478 PMC6767378

[8] Woodhead MM & Baddeley AD (1981). Individual differences and memory for faces, pictures, and words. Mem Cognit: 9, 368–370. 10.3758/bf03197561 7278625

[9] Megreya AM & Burton AM (2006). Unfamiliar faces are not faces: Evidence from a matching task. Mem & Cognit: 34, 865–876. 10.3758/BF03193433 17063917

[10] Bruce V, Henderson Z, Greenwood K, Hancock PJB, Burton AM et al (1999). Verification of face identities from images captured on video. J Exp Psychol Appl: 5, 339–360. 10.1037//1076-898X.5.4.339

[11] Burton AM, White D & McNeill A (2010). The Glasgow Face Matching Test. Behav Res Methods: 42, 286–291. 10.3758/BRM.42.1.286 20160307

[12] Bindemann M, Avetisyan M & Rakow T (2012). Who can recognize unfamiliar faces? Individual differences and observer consistency in person identification. J Exp Psychol Appl: 18, 277–91. 10.1037/a0029635 22905851

[13] Wilhelm O, Herzmann G, Kunina O, Danthiir V, Schacht A & Sommer W (2010). Individual differences in perceiving and recognizing faces—One element of social cognition. J Pers Soc Psychol: 99, 530–48. 10.1037/a0019972 20677889

[14] Wilmer JB, Germine L, Chabris CF, Chatterjee G, Williams M, Loken E, et al (2010). Human face recognition ability is specific and highly heritable. Proc Natl Acad Sci U S A: 16, 107, 5238–41. 10.1073/pnas.0913053107 20176944 PMC2841913

[15] Kokje E, Bindemann M, & Megreya AM (2018). Cross-race correlations in the abilities to match unfamiliar faces. Acta Psychol: 185,13–21. 10.1016/j.actpsy.2018.01.006 29407241

[16] Megreya AM & Bindemann M (2009). Revisiting the processing of internal and external features of unfamiliar faces: The headscarf effect. Perception: 38, 1831–1848. 10.1068/p6385 20192132

[17] Megreya AM & Bindemann M (2018). Feature instructions improve face-matching accuracy. PLoS One: 15,13(3):e0193455 10.1371/journal.pone.0193455 29543822 PMC5854257

[18] Megreya AM, White D & Burton AM (2011). The other race effect does not rely on memory: Evidence from a matching task. Q J Exp Psychol: 64, 1473–1483. 10.1080/17470218.2011.575228 21812594

[19] Germine L. T., Duchaine B., & Nakayama K. (2011). Where cognitive development and aging meet: Face learning ability peaks after age 30. *Cognition*, 118, 201–210 10.1016/j.cognition.2010.11.002 21130422

[20] Megreya AM & Bindemann M (2015). Developmental improvement and age-related decline in unfamiliar face matching. Perception: 44, 5–22. 10.1068/p7825 26489213

[21] Lewin C & Herlitz A (2002). Sex differences in face recognition—Women's faces make the difference. Brain Cogn: 50,121–8. 10.1016/s0278-2626(02)00016-7 12372357

[22] Megreya AM & Bindemann M (2012). Identification accuracy for single- and double-perpetrator crimes: Does accomplice gender matter? Br J Psychol: 103, 439–53. 10.1111/j.2044-8295.2011.02084.x 23034106

[23] Megreya AM, Bindemann M & Havard C (2011). Sex differences in unfamiliar face identification: Evidence from matching tasks. Acta Psychol:137, 83–9. 10.1016/j.actpsy.2011.03.003 21459354

[24] Rehnman J & Herlitz A (2006). Higher face recognition ability in girls: Magnified by own-sex and own-ethnicity bias. *Memory*, 14, 289−296. 10.1080/09658210500233581 16574585

[25] Verhallen RJ, Bosten JM, Goodbourn PT, Lawrance-Owen AJ, Bargary G & Mollon JD (2017). General and specific factors in the processing of faces. *Vision Res*, 141, 217–227. 10.1016/j.visres.2016.12.014 28077292

[26] McCaffery JM, Robertson DJ, Young AW, & Burton AM. (2018). Individual differences in face identity processing. Cogn Res Princ Implic: 3:21 10.1186/s41235-018-0112-9 30009251 PMC6019420

[27] Robertson DJ, Black J, Chamberlain B, Megreya AM & Davis JP (2020). Super-recognisers show an advantage for other race face identification. Appl Cognitive Psych, 34, 205–216. 10.1002/acp.3608

[28] Stacchi L, Huguenin-Elie E, Caldara R, & Ramon M (2020). Normative data for two tests of face matching under ecological conditions. Cogn Res Princ Implic: 5(1):8 10.1186/s41235-019-0205-0 32076893 PMC7031457

[29] Megreya AM & Burton AM (2007). Hits and false positives in face matching: A familiarity-based dissociation. Percept Psychophys: 69, 1175–1184. 10.3758/bf03193954 18038955

[30] Bindemann M, Brown C, Koyas T & Russ A (2012). Individual differences in face identification postdict eyewitness accuracy. J. Appl. Res. Mem. Cogn, 1, 96–103. 10.1016/j.jarmac.2012.02.001

[31] Xu B, Liu-Shuang J, Rossion B, & Tanaka J (2017). Individual differences in face identity processing with fast periodic visual stimulation. J Cogn Neurosci: 29,1368–1377. 10.1162/jocn_a_01126 28358660

[32] Dennett HW, McKone E, Tavashmi R, Hall A, Pidcock M, Edwards M, et al (2012). The Cambridge Car Memory Test: A task matched in format to the Cambridge Face Memory Test, with norms, reliability, sex differences, dissociations from face memory, and expertise effects. Behav Res Methods: 44,587–605. 10.3758/s13428-011-0160-2 22012343

[33] Rose SA, Feldman JF & Jankowski JJ (2003). Infant visual recognition memory: Independent contributions of speed and attention. Dev Psychol: 39, 563–71. 10.1037/0012-1649.39.3.563 12760523

[34] Rose SA & Feldman JF (1995). Prediction of IQ and specific cognitive abilities at 11 years from infancy measures. Dev. Psychol, 31, 685–696. 10.1037/0012-1649.31.4.685

[35] Kanwisher N (2000). Domain specificity in face perception. Nat Neurosci: 3,759–63. 10.1038/77664 10903567

[36] Li J, Tian M, Fang H, Xu M, Li H & Liu J (2010). Extroversion predicts individual differences in face recognition. Commun Integr Biol: 3, 295–8. 10.4161/cib.3.4.12093 20798810 PMC2928302

[37] Saito T, Nakamura T & Endo T (2005). Big five personality factors related to face recognition. *Jap J*. *Psychol*: 75, 517–522. 10.4992/jjpsy.75.517 15782589

[38] Cheung CHM, Rutherford HJV, Mayes LC, & McPartland JC. (2010). Neural responses to faces reflect social personality traits. Soc Neurosci: 5, 351–359. 10.1080/17470911003597377 20544557 PMC3075017

[39] Bothwell RK, Brigham JC & Pigott MA (1987). An exploratory study of personality differences in eyewitness memory J Soc Behav Pers: 2, 335–343.

[40] Pozzulo JD, Crescini C, Lemieux JMT, & Tawfik A (2007). The effect of shyness on eyewitness memory and the susceptibility to misinformation. Pers Individ Dif: 43, 1656–1666. 10.1016/j.paid.2007.05.001

[41] Thompson WB & Mueller JH (1984). Face Memory and Hemispheric Preference: Emotionality and Extroversion. Brain & Cogn: 3, 239–248. 10.1016/0278-2626(84)90019-8 6536328

[42] Trouvé RJ & Libkuman TM (1992). Eyewitness performance of personality types as a function of induced arousal. Am J Psychol: 105, 417–433. 10.2307/1423196

[43] Ward RA & Loftus EF (1985). Eyewitness performance in different psychological types. *The* J Gen Psychol: 112,191–200. 10.1080/00221309.1985.9711003 4056764

[44] Megreya AM & Bindemann M (2013). Individual differences in personality and face identification. *J Cogn Psychol*: 25, 30–37. 10.1080/20445911.2012.739153

[45] Lander K & Poyarekar S (2015). Famous face recognition, face matching, and extraversion. Q J Exp Psychol: 68,1769–1776. 10.1080/17470218.2014.988737 25811985

[46] Brigham JC, Maass A, Martinez D & Whittenberger G (1983). The effect of arousal on facial recognition. Basic Appl Soc Psychol: 4, 279–293. 10.1207/s15324834basp0403_6

[47] Hills PJ, Dickinson D, Daniels LM, Boobyer CA & Burton R (2019). Being observed caused physiological stress leading to poorer face recognition. Acta Psychol: 196, 118–128. 10.1016/j.actpsy.2019.04.012 31054376

[48] Deffenbacher KA, Bornstein BH, Penrod SD & McGorty K (2004). A meta-analytic review of the effects of high stress on eyewitness memory. Law Hum Behav: 28, 687–706. 10.1007/s10979-004-0565-x 15732653

[49] Mueller JH, Bailis KL & Goldstein AG (1979). Depth of processing and anxiety in facial recognition. Br J Psychol:70, 511–515. 10.1111/j.2044-8295.1979.tb01724.x 509018

[50] Nowicki S, Winograd E & Millard BA (1979). Memory for faces: A social learning analysis. J Res Pers: 13, 460–468. 10.1016/0092-6566(79)90008-4

[51] Valentine T & Mesout J (2009). Eyewitness identification under stress in the London Dungeon. App Cogn Psychol, 23, 151–161. 10.1002/acp.1463

[52] Bobak AK, Pampoulov P, & Bate S (2016). Detecting superior face recognition skills in a large sample of young British adults. Front Psychol: 22, 7:1378 10.3389/fpsyg.2016.01378 27713706 PMC5031595

[53] Davis JM, McKone E, Dennett H, O’Connor KB, O’Kearney R & Palermo R et al (2011) Individual differences in the ability to recognise facial identity are associated with social anxiety. PLoS ONE: 6(12): e28800 10.1371/journal.pone.0028800 22194916 PMC3237502

[54] Bate S, Parris B, Haslam C & Kay J (2010). Socio-emotional functioning and face recognition ability in the normal population. Pers Individ Dif: 48, 239–242. 10.1016/j.paid.2009.10.005

[55] Gross JJ (2014). Emotion regulation: conceptual and empirical foundations In Gross JJ (Ed) Handbook of Emotion Regulation (pp. 3–20), second edition New York, NY: The Guilford Press.

[56] Kappas A (2011). Emotion and regulation are one! EMR: 3, 17–25. 10.1177/1754073910380971

[57] Thompson RA (2011). Emotion and Emotion Regulation: Two Sides of the Developing Coin. EMR, 1, 53–61. 10.1177/1754073910380969

[58] Gross JJ (1998). Antecedent- and response-focused emotion regulation: Divergent consequences for experience, expression, and physiology. J Pers Soc Psychol: 74, 224–37. 10.1037//0022-3514.74.1.224 9457784

[59] Gross JJ (2001). Emotion regulation in adulthood: Timing is everything. Curr Dir Psychol Sci: 10, 214–219. 10.1111/1467-8721.00152

[60] Garnefski N, Kraaij V & Spinhoven P (2001). Negative life events, cognitive emotion regulation, and emotional problems. Pers Individ Dif: 30, 1311–1327. 10.1016/S0191-8869(00)00113-6

[61] Aldao A, Nolen-Hoeksema S & Schweizer S (2010). Emotion-regulation strategies across psychopathology: a meta-analytic review. Clin Psychol Rev: 30, 217–37. 10.1016/j.cpr.2009.11.004 20015584

[62] Richards JM & Gross JJ (2000). Emotion regulation and memory: The cognitive costs of keeping one’s cool. J Pers Soc Psychol:79, 410–24. 10.1037//0022-3514.79.3.410 10981843

[63] Marceau E, Kelly P & Solowij N (2018). The relationship between executive functions and emotion regulation in females attending therapeutic community treatment for substance use disorder. Drug Alcohol Depend:182, 58–66. 10.1016/j.drugalcdep.2017.10.008 29154148

[64] McRae K, Jacobs SE, Ray RD, John OP & Gross JJ (2012). Individual differences in reappraisal ability: Links to reappraisal frequency, well-being, and cognitive control. J Res Pers: 46, 2–7. 10.1016/j.jrp.2011.10.003

[65] Schmeichel BJ, Volokhov RN & Demaree HA (2008). Working memory capacity and the self-regulation of emotional expression and experience. J Pers Soc Psychol: 95,1526–1540. 10.1037/a0013345 19025300

[66] Schweizer S, Grahn J, Hampshire A, Mobbs D & Dalgleish T (2013). Training the emotional brain: Improving affective control through emotional working memory training. J Neurosci: 33, 5301–5311. 10.1523/JNEUROSCI.2593-12.2013 23516294 PMC6704999

[67] Xiu L, Zhou R & Jiang Y (2016).Working memory training improves emotion regulation ability: Evidence from HRV. Physiol Behav: 155, 25–29. 10.1016/j.physbeh.2015.12.004 26679738

[68] Xiu L, Wu J, Chang L & Zhou R (2018). Working Memory Training Improves Emotion Regulation Ability. Sci Rep: 8,15012 10.1038/s41598-018-31495-2 30301906 PMC6177433

[69] Druzgal TJ & D’esposito M (2001a). Activity in fusiform face area modulated as a function of working memory load. Brain Res Cogn Brain Res: 10, 355–364. 10.1016/s0926-6410(00)00056-2 11167061

[70] Druzgal TJ & D’esposito M (2003). Dissecting contributions of prefrontal cortex and fusiform face area to face working memory. J Cogn Neurosci: 15, 771–784. 10.1162/089892903322370708 14511531

[71] Jackson MC & Raymond JE (2008).Familiarity enhances visual working memory for faces. J Exp Psychol Hum Percept Perform: 34, 556–568. 10.1037/0096-1523.34.3.556 18505323 PMC4262787

[72] Megreya AM & Burton AM (2008). Matching faces to photographs: Poor performance in eyewitness memory (without the memory). J Exp Psychol Appl: 14, 364–372. 10.1037/a0013464 19102619

[73] Megreya AM, Memon A & Havard C (2012). The headscarf effect: Direct evidence from eyewitness identification paradigm. App Cogn Psychol: 26, 308–315. 10.1002/acp.1826

[74] Gross JJ & John OP (2003). Individual differences in two emotion regulation processes: Implications for affect, relationships, and well-being. J Pers Soc Psychol: 85, 348–62. 10.1037/0022-3514.85.2.348 12916575

[75] Megreya AM, Latzman RD, Al-Emadi AA & Al-Attiyah AA (2018). An integrative model of emotion regulation and associations with positive and negative affectivity across four Arabic speaking countries and the USA. *Motiv Emot*: 42, 566–575. 10.1007/s11031-018-9682-6

[76] Butler EA, Egloff B, Wlhelm FH, Smith NC, Erickson EA & Gross JJ (2003). The social consequences of expressive suppression. *Emotion*, 3, 48–67. 10.1037/1528-3542.3.1.48 12899316

[77] Megreya AM, Sandford A & Burton AM (2013). Matching face images taken on the same day or months apart: The limitations of photo-ID. Appl Cognitive Psych: 27, 700–706. 10.1002/acp.2965

[78] White D, Kemp RI, Jenkins R & Burton AM (2014). Feedback training for facial image comparison. Psychon B Rev: 21, 100–106. 10.3758/s13423-013-0475-3 23835616

[79] Megreya AM, Latzman RD, Al-Attiyah AA & Alrashidi M (2016). The robustness of the Nine-Factor Structure of the Cognitive Emotion Regulation Questionnaire across Four Arabic Speaking Middle Eastern Countries. J Cross-Cult Psychol: 47, 875–890. 10.1177/0022022116644785

[80] Garnefski N, Kraaij V & Spinhoven P (2002). Manual for the use of the Cognitive Emotion Regulation Questionnaire: A questionnaire measuring cognitive coping strategies. Leiderdorp, The Netherlands, DATEC.

[81] Kivity Y & Huppert J D (2019). Emotion regulation in social anxiety: a systematic investigation and meta-analysis using self-report, subjective, and event-related potentials measures. Cogn Emot: 33, 213–230. 10.1080/02699931.2018.1446414 29514588

[82] Garnefski N & Kraaij V (2007). The cognitive emotion regulation questionnaire. psychometric features and prospective relationships with depression and anxiety in adults. Eur J Psychol Assess: 23, 141–149. 10.1027/1015-5759.23.3.141

[83] Leigh E & Clark D M (2018). Understanding social anxiety disorder in adolescents and improving treatment outcomes: Applying the cognitive model of Clark and Wells (1995). Clin Child Fam Psychol Rev: 21, 388–414. 10.1007/s10567-018-0258-5 29654442 PMC6447508

[84] Mobini S, Reynolds S & Mackintosh B (2013). Clinical implications of cognitive bias modification for interpretative biases in social anxiety: An integrative literature review. Cogn Ther Res: 37, 173–182. 10.1007/s10608-012-9445-8

[85] Pessoa L (2008). On the relationship between emotion and cognition. Nat Rev Neurosci: 9,148–58. 10.1038/nrn2317 18209732

[86] Blair KS, Smith BW, Mitchell DGV, Morton J, Vythilingam M, et al, (2007). Modulation of emotion by cognition and cognition by emotion. Neuroimage, 35, 430–440. 10.1016/j.neuroimage.2006.11.048 17239620 PMC1862681

[87] Megreya AM (2018). Feature-by-feature comparison and holistic processing in unfamiliar face matching. *PeerJ*, 6, e4437 10.7717/peerj.4437 29503772 PMC5831152

[88] Towler A, White D & Kemp RI (2017). Evaluating the feature comparison strategy for forensic face identification. J Exp Psychol Appl: 23, 47–58. 10.1037/xap0000108 28045276

[89] Kraaij V & Garnefski N (2019). The Behavioral Emotion Regulation Questionnaire: Development, psychometric properties and relationships with emotional problems and the Cognitive Emotion Regulation Questionnaire. Pers Individ Dif: 137, 56–61. 10.1016/j.paid.2018.07.036

[90] Gratz K L & Roemer L (2004). Multidimensional assessment of emotion regulation and dysregulation: Development, factor structure, and initial validation of the difficulties in emotion regulation scale. J Psychopath Behav Assess: 26, 41–54. 10.1023/B:JOBA.0000007455.08539.94

