# Supplementary material for: Individual differences in emotion regulation and face recognition
Source: PLoS One. 2020 Dec 10;15(12):e0243209. doi: 10.1371/journal.pone.0243209 (PMC7728238; doi:10.1371/journal.pone.0243209)
Supplement: S1 File — (DOCX) [file pone.0243209.s002.docx]

**S1 File.**

The Simple Main Effects of overall accuracy and false positives

**Simple Main Effects for overall accuracy**

| Source of Variation | Sum of Squares | Degree of Freedom | Mean Squares | F | P |
| --- | --- | --- | --- | --- | --- |
| Nationality at |  |  |  |  |  |
| Same | 384.62 | 1 | 384.62 | 7.355 | 0.0091 |
| Different | 2261.78 | 1 | 2261.78 | 43.251 | 0.0000 |
| Error Term | 2667.03 | 51 | 52.295 |  |  |
| Image time at |  |  |  |  |  |
| Egyptian | 569.18 | 1 | 569.18 | 9.444 | 0.0034 |
| UK | 2683.66 | 1 | 2683.66 | 44.53 | 0.0000 |
| Error Term | 3073.8 | 51 | 60.271 |  |  |

**Simple Main Effects for false positives**

| Source of Variation | Sum of Squares | Degree of Freedom | Mean Squares | F | P |
| --- | --- | --- | --- | --- | --- |
| Nationality at |  |  |  |  |  |
| Same | 20.94 | 1 | 20.94 | 0.121 | 0.729 |
| Different | 4446.15 | 1 | 4446.15 | 25.72 | 0.0000 |
| Error Term | 8816.94 | 51 | 172.88 |  |  |
| Image_time at |  |  |  |  |  |
| Egyptian | 649.83 | 1 | 649.83 | 4.465 | 0.037 |
| UK | 1340.41 | 1 | 1340.41 | 9.415 | 0.003 |
| Error Term | 7260.79 | 51 | 142.37 |  |  |
